# Trabecular bone deterioration in differentiated thyroid cancer: Impact of long‐term TSH suppressive therapy

**DOI:** 10.1002/cam4.3200

**Published:** 2020-06-25

**Authors:** Federico Hawkins Carranza, Sonsoles Guadalix Iglesias, María Luisa De Mingo Domínguez, Cristina Martín‐Arriscado Arroba, Begoña López Álvarez, Gonzalo Allo Miguel, Guillermo Martínez Díaz‐Guerra

**Affiliations:** ^1^ Research Institute i+12 University Hospital 12 de Octubre Madrid Spain; ^2^ Thyroid Cancer Unit Service of Endocrinology University Hospital 12 de Octubre Madrid Spain; ^3^ Hospital La Luz Madrid Spain; ^4^ Epidemiology Unit Research Institute i+12 University Hospital 12 de Octubre Madrid Spain; ^5^ Centro de Salud Goya Madrid Spain; ^6^ Service of Endocrinology University Hospital 12 de Octubre Madrid Spain

**Keywords:** bone mineral density, differentiated thyroid carcinoma, thyrotropin suppressive therapy, trabecular bone score

## Abstract

**Background:**

Conflicting results has been reported regard osteoporosis and fractures in patients with Differentiated Thyroid Cancer (DTC). Our objective was to evaluate the long‐term effects of TSH suppression therapy with Levothyroxine (LT4) on trabecular bone score (TBS) and bone mineral density (BMD) in females with DTC after thyroidectomy.

**Methods:**

About 145 women with resected DTC and receiving long‐term TSH therapy, were stratified according to the degree of TSH suppression. Mean duration of follow‐up was 12.3 ± 6.1 years. BMD and TBS, were assessed using dual‐energy X‐ray absorptiometry (DXA) and TBS iNsight (Med‐Imaps), at baseline (1‐3 months after surgery) and at the final study visit.

**Results:**

In patients stratified by duration of TSH suppression therapy (Group I, 5‐10 years; Group II, >10 years), slight increases from baseline TSH levels were observed. Significant decreases in LS‐BMD and FN‐BMD were seen in patients after >10 years. TBS values were lower in Groups I (1.289 ± 0.122) and II (1.259 ± 0.129) compared with baseline values (*P* = .0001, both groups). Regarding the degree of TSH suppression, TBS was significantly reduced in those with TSH < 0.1 µU/mL (*P* = .0086), and not in patients with TSH suppression of 0.1.‐0.5 or >0.5 µU/mL.

**Conclusions:**

We found deterioration of trabecular structure in patients with DTC and TSH suppression therapy below 0.1 µU/mL and after 5‐10 years of follow‐up. Significant changes in BMD according to TSH levels were not observed. Trabecular Bone Score is a useful technique for identifying thyroid cancer patients with risk of bone deterioration.

## Graphical abstract

There is a debate respect the effect of long‐term TSH suppression therapy in Thyroid Cancer. We have demonstrated according to the degree of suppression and duration (Group I, 5‐10 years; Group II, >10 years): TBS values were lower in Groups I (1.29 ± 0.12) and II (1.26 ± 0.13) compared with baseline values while decreases in L‐BMD and FN‐BMD were seen in patients after >10 years. Regarding the degree of TSH suppression, TBS was significantly reduced in those with TSH < 0.1 µU/mL (*P* = .0086), and not in patients with TSH suppression of 0.1.‐0.5 or >0.5 µU/mL. We described for the first time deterioration of trabecular structure in patients with DTC and TSH suppression therapy below 0.1 µU/mL and after 5‐10 years of follow‐up. Significant changes in BMD according to TSH levels were not observed. Therefore we support Trabecular Bone Score as a useful technique for identifying thyroid cancer patients with risk of bone fracture.

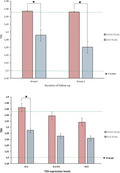

## 1 INTRODUCTION

The incidence of thyroid cancer has been described as increasing worldwide in the last decades.^1^ The mainstay of treatment of patients with differentiated thyroid cancer (DTC) is surgery. Thereafter, TSH suppression with levothyroxine (LT4) is recommended as a main therapeutic option for patients with DTC, to prevent tumor recurrence and increase survival.^2^ American Thyroid Association (ATA) 2016 guidelines for the use of thyroid hormone therapy in DCT recommend TSH level targets based on a patient's risk of recurrence: 0.1 µU/mL for high‐risk patients; 0.1‐0.5 µU/L for intermediate‐risk patients; and 0.5‐2 µU/mL for low‐risk patients, who have undergone remnant ablation and have undetectable serum thyroglobulin levels.^3^

Although meta‐analysis has confirmed that patients with DTC and TSH suppression showed a significant reduced risk of disease progression, recurrence, and death (relative risk [RR] = 0.73, 95% CI = 0.60‐0.88, *P* < .05),^4^ recent studies have found no significant benefit t4regarding disease‐specific or disease‐free survival, in DTC patients with undetectable serum TSH levels versus subnormal TSH levels, when also taking in consideration the degree of low‐risk of thyroid cancer.^5^, ^6^ Further, because DTC usually is an indolent tumor, and its mortality rate is very low, TSH suppression with LT4 can be controversial, given that treatment can induce a state of iatrogenic subclinical hyperthyroidism, which can be associated with bone and cardiovascular adverse effects.

There is controversy about the association between fracture risk and subclinical hyperthyroidism, either of endogenous cause or related to thyroxine treatment. Patients with LT4 treatment and TSH suppression levels, followed for 8 years, were not associated to any increase fractured rate; however, there was an increased risk of ischemic heart disease.^7^ In the first Cardiovascular Health Study (CHS) no association between endogenous subclinical hyperthyroidism and hip fracture in women was found.^8^ Data from the CHS has been recently enlarged, confirming no association between endogenous subclinical hyperthyroidism and an increased risk of hip fracture or lower BMD at the spine or hip in a study with 5888 elderly subjects.^9^ In the contrary, an observational study showed that patients taking thyroid medication, with high TSH and those with a suppressed TSH, were both at increased risk of fracture, suggesting that hyperthyroxinemia together with suppressed TSH could detrimental to bone.^10^ A meta‐analysis of 13 prospective cohorts’ studies showed that endogenous subclinical hyperthyroidism was associated with HRs of 1.36 (95% CI, 1.13‐1.64) for hip fracture, and 1.28 (95% CI, 1.06‐1.53) for any fracture, while in the comparison between participants treated with LT4 versus untreated patients, therapy with LT4 was not associated with any fracture outcomes 0.98 (95% CI, 0.82‐1.17).^11^ A recently meta‐analysis with 24 studies, confirm this results, and extends this effect on fracture risk at various sites and to lower distal and ultradistal BMD.^12^

In particular, long‐term TSH suppression therapy has been called into question due to its association with increased bone loss and fracture incidence.^13^ Recently, in a large study, compared with controls, osteoporosis, but not fractures was more frequent in patients with thyroid cancer (OR 1.33; 95%CI 1.18‐1.49).^14^

Although several studies have not found an association between bone loss and TSH suppressive therapy in men and premenopausal women with DTC, a substantial number of other studies in postmenopausal women with DTC have identified this adverse effect.^15^ The discrepancy in these studies could be explained by the heterogeneity of the patient populations included, the degree and duration of TSH suppression, or the methods used to measure bone density and quality. Also, because the number of thyroid cancer survivors is growing, more patients may experience the long‐term effect of TSH suppression therapy, and is mandatory to develop methods for the accurately identification of patients at risk of osteoporosis and fractures.

Dual‐energy X‐ray absorptiometry (DXA) for measurement of bone mineral density (BMD), can be now performed together with Trabecular Bone Scores (TBS) analysis. TBS is a gray‐level texture measure derived from lumbar spine DXA images. It is an indirect measure of trabecular microarchitecture, which gives additional information regarding bone quality, that can be useful for patients with risk factors for bone loss, including those under TSH suppression therapy.^10^ Longitudinal studies have shown that TBS predicts fracture risk in women, even after adjusting for BMD.^16^ The use of both measurements, TBS plus BMD, also improves fracture discrimination.^17^

The aim of this study was to use TBS and DXA to assess the effect of the degree and duration of long‐term TSH suppression therapy, on bone microarchitecture and BMD in female patients with DTC after total thyroidectomy who were treated in our Thyroid Cancer Unit.

## 2 MATERIAL AND METHODS

### 2.1 Data and study population

In this study, inclusion criteria were women with DTC who received total thyroidectomy with ^131^I ablation, when necessary, and who received long‐term TSH suppressive therapy with LT4 according to guidelines.^2^ Patients were required to initiate TSH suppression immediately after surgery, have blood extraction and a DXA scan 1‐3 months after surgery, and have a follow‐up period of ≥5 years. The exclusion criteria were as follow: (a) the use of medications that might affect bone metabolism including estrogen/progestin, glucocorticoids, bisphosphonate, calcitonin, selective estrogen receptor modulators, denosumab, teriparatide, and lithium; (b) malabsorption syndrome; (c) diuretics; (d) diseases affecting bone metabolism (eg, Paget's bone disease, renal osteodystrophy), malignant neoplasms, hyperparathyroidism, primary and postsurgical hypoparathyroidism and hyperthyroidism. Patients without a complete set of data were also excluded. Men were not included in the analysis.

To study the effect of the long‐term TSH suppression therapy, the included cohort was stratified at the final visit according to the degree of suppression of TSH: suppressed (<0.1 µU/mL), moderately suppressed (0.1‐0.5 µU/mL) and nonsuppressed (>0.5 µU/mL). Patients were also surveyed to assess risk factors for low bone mass, as smoking, daily calcium intake, and physical activity. Other clinical data were retrieved using the information collected in patient files. Body mass index (BMI) was calculated as the weight in kilograms divided by the height in meters squared. Time of follow‐up was calculated from the start of TSH suppression therapy, which was initiated immediately after total thyroidectomy, to the final study visit. Diagnosis, surgery, and follow‐up of all patients occurred at the Thyroid Cancer Unit of our Hospital. Ethical approval for this study was obtained from our Institutional Review Board prior to beginning this study. Informed consent was obtained from all enrolled patients.

### 2.2 Biochemical analysis

Blood extraction were obtained 1‐3 months postoperatively and at the final study visit. Serum samples for biochemical analyses were obtained between 8 and 9 am after overnight fast and immediately kept frozen at −70°C until they were measured by auto analyzer (Modular P800 Chemistry Analyzer, Roche Diagnostic). Serum levels of creatinine, calcium (corrected for albumin binding), and phosphate were measured. Serum TSH (Architect TSH reagent; Abbot Laboratories) and free (T4) by electrochemiluminescence (ElecsysT4, Roche Diagnostic; functional sensitivity <0.01 µg/mL).

### 2.3 Assessment of BMD and TBS

DXA scans were performed within 1‐3 months of thyroidectomy and at the final study visit. BMD was measured by Dual X‐ray absorptiometry (DXA, densitometer QDR 4500, Hologic, Waltham MA, USA) at lumbar spine, L1‐L4, ( LS‐BMD), femoral neck (FN‐BMD), total hip (TH‐BMD), ultradistal radius (UDR‐BMD), total radius (TR‐BMD), and distal third of the radius (1/3 DR‐BMD). The same equipment was used during the entire study. The coefficient of variation was 0.95% at the LS‐BMD and 2.1% at FN‐BMD. BMD values are expressed as grams per square centimeter (g/cm^2^) which is expressed from the expected peak young‐ adult mean BMD for the T scores. According to the WHO criteria, patients were classified as osteoporotic (T score equal or worse than −2.5), osteopenic (T score −1≥ and >−2.5), and normal (T score > −1).^18^ Reference data corresponding to the Spanish population were obtained from a multicenter study with 2442 healthy subjects, aged 20‐80 years.^19^

TBS measurements were performed applying the TBS iNsight2.0 software (Med‐Imaps, Geneva, Switzerland) to the LS DXA exams. Lumbar TBS was calculated as the mean value of individual measurements for vertebrae L1‐L4. Weight and height of each patient are entered in the software program in each visit corresponding to the TBS study.

Reference values are: normal (TBS ≥ 1.35); is considered normal; partially degraded microarchitecture (TBS > 1.20 and 1.35); and degraded microarchitecture (≤1.20).^20^ The coefficient of variation of TBS calculated from three repeated measurements in 15 women was 0.8%.

### 2.4 Statistical analysis

All data were analyzed using SAS statistical package (version 9.3; SAS Institute). Continuous variables were expressed as the mean ± standard deviation (SD). Normality of data was confirm using the Kolmogorov‐Smirnov test. Category variables were expressed by their absolute and relative percentage, and analyzed using contingence tables and Chi‐square or Fischer test. The Wilcoxon nonparametric test or Kruskal‐Wallis nonparametric test were used for the analysis of more than two parameters in the transversal study, and the Student´s t test for the longitudinal study. The Pearson test was used to evaluate the relationship between bone parameters and duration and TSH suppression level. Fisher's exact test was used to study the significance of the association observed in the categorical data. Multiple lineal regressions was performed to evaluate the dependence and influence between TBS and other variables. All analyzes were adjusted for follow‐up time. A level of *α* = .05 was considered significant in all statistical procedures. The Bonferroni test was used in the correction of multiple comparison tests.

## 3 RESULTS

### 3.1 Description of sample

A total of 145 Caucasian women (131 postmenopausaland 14 premenopausal) with DTC were included in this study. Clinical and bone densitometry data at baseline and at the final study visit are shown in Table 1. The mean follow‐up with LT4 suppression therapy after total thyroidectomy was 12 years, with a range of 7‐20 years, and the mean age of women at the end of the study was 64 ± 10.6 years. Mean BMI was higher in patients at the end of the study (28.45 ± 5.3 kg/m^2^), compared to baseline (27.27 ± 0.6 kg/m^2^; *P* < .0001). At the end of the study, there was a significant decrease from baseline in prescribed LT4 doses from 2.29 ± 0.6 µg/kg to 1.70 ± 0.4 µg/kg (*P* = .0417) and a significant increase in TSH levels from 0.23 ± 0.4 µU/mL to 0.89 ± 0.1 µU/mL (*P* < .0001).

**TABLE 1:** Clinical and bone densitometry characteristics of DTC patients included in the study at baseline and end studies

Studied parameters	Baseline study (n = 145)	End study (n = 145)	*P* value
Clinical and hormonal data			
Age (years)	51.48 ± 11.9	63.96 ± 10.65	<.0001
IMC (kg/m^2^)	27.27 ± 0.6	28.45 ± 5.3	<.0001
LT4 doses (mcg/Kg)	2.29 ± 0.6	1.70 ± 0.4	.0417
Serum free T4 (ng/dL)	1.64 ± 0.4	1.64 ± 0.3	.9464
Serum TSH (µU/mL)	0.23 ± 0.4	0.89 ± 0.1	<.0001
Duration‐years (range)	—	12.3 ± 6.1 (7‐20)	
Bone densitometry			
LS‐BMD g/cm^2^	0.91 ± 0.16	0.89 ± 0.13	.1122
FN‐BMD g/cm^2^	0.74 ± 0.14	0.70 ± 0.11	.0635
TH‐BMD g/cm^2^	0.84 ± 0.11	0.86 ± 0.13	.5102
UDR‐BMD g/cm^2^	0.42 ± 0.06	0.40 ± 0.06	.3132
1/3 DR‐BMD g/cm^2^	0.62 ± 0.05	0.62 ± 0.08	.9232
TR‐BMD g/cm^2^	0.52 ± 0.05	0.50 ± 0.07	.1857
LS‐T score	−1.23 ± 1.3	−1.40 ± 1.2	.1656
FN‐T score	−1.28 ± 1.3	−1.35 ± 1.0	.7040
TH‐ T score	−0.89 ± 0.9	−0.68 ± 1.0	.2552
UDR‐T score	−0.47 ± 0.9	−0.85 ± 1.1	.3254
1/3DR‐T score	−1.24 ± 0.8	−1.22 ± 1.2	.9442
TR‐T score	−0.97 ± 0.7	−1.55 ± 1.2	.1359
TBS	1.35 ± 0.14	1.27 ± 0.13	<.0001

Note

Highlighted red values indicate statically significant values.

Abbreviations: 1/3 DR, 1/3 distal radius; BMD, bone mineral density; FN, femoral neck; LS, lumbar spine; TH, total hip; TR, total radius; UDR, ultradistal radius.

No significant changes were observed in areal BMD or T score at all skeletal sites. In contrast, TBS decreased from 1.346 ± 0.136 (normal range) to 1.273 ± 0.136 (partially degraded) (*P *< .0001). Estimated mean dietary calcium intake of DTC patients (575.94 ± 282 mg/d), was only collected at final study visit, and no differences were observed among patients with normal BMD (561 ± 195 mg/d), osteopenia (572 ± 301 mg/d), and osteoporosis (588 ± 294 mg/d).

### 3.2 Stratification according to years of follow‐up

Table 2 shows the results of the final visit stratified by the number of years of follow‐up: Group I (n = 69) includes patients with a follow‐up duration of 5‐10 years and Group II (n = 76), includes patients with >10 years of follow‐up. As expected, there were also significant changes in BMI at the end of the study in both groups, compared to baseline (Group I, end of study 28.67 ± 5.9 kg/m^2^ vs 27.77 ± 5.6 kg/m^2^, *P* = .0042; Group II, end of study 28.41 ± 4.8 kg/m^2^ vs 26.87 ± 4.0 kg/m^2^, *P* = .0009). Both groups also had increases in TSH levels (from 0.17 ± 0.36 µU/ml to 0.76 ± 1.6 µU/mL in Group 1 [*P* = .0027], and from 0.27 ± 0.5 µU/mL to 1.12 ± 1.85 in Group II [*P* = .0002]). There was also a significant decrease in mean LT4 doses in both groups (Group I from 2.10 ± 0.5 µg/kg to 1.71 ± 0.3 µg/kg, *P* < .0001; Group II from 2.47 ± 0.62 µg/kg to 1.69 ± 0.50 µg/kg, *P* < .0001).

**TABLE 2:** Study of patients with differential thyroid carcinoma according to duration of follow‐up

Years follow‐up (n)	5‐10 years Group I (n = 69)			>(10 years Group II (n = 76)		
Period of study	Baseline study	End study	*P*	Baseline study	End study	*P*
Age (years)	54.55 ± 11.4	62.44 ± 11.3	<.0001	47.64 ± 10.34	64.86 ± 9.58	<.0001
BMI (kg/m^2^)	27.77 ± 5.6	28.67 ± 5.9	.0042	26.87 ± 4.0	28.41 ± 4.8	.0009
LT4 doses (mcg Kg)	2.10 ± 0.5	1.71 ± 0.3	<.0001	2.47 ± 0.62	1.69 ± 0.50	<.0001
Serum free T4 (ng/dL)	1.53 ± 0.3	1.64 ± 0.3	.0143	1.75 ± 0.5	1.62 ± 0.25	.0499
Serum TSH (µU/mL)	0.17 ± 0.36	0.76 ± 1.6	.0027	0.27 ± 0.5	1.12 ± 1.85	.0002
Duration‐years (range)	—	7.5 ± 1.8 (6‐9)		—	17.5 ± 4.1 (13‐21)	
LS‐BMD g/cm^2^	0.90 ± 0.13	0.91 ± 0.13	.2569	0.91 ± 0.18	0.87 ± 0.13	.0249
FN‐BMD g/cm^2^	0.68 ± 0.13	0.71 ± 0.12	.8026	0.78 ± 0.14	0.69 ± 0.11	<.0001
TH‐BMD g/cm^2^	0.82 ± 0.09	0.87 ± 0.13	.0721	0.86 ± 0.13	0.85 ± 0.14	.3053
UDR‐BMD g/cm^2^	0.42 ± 0.06	0.41 ± 0.07	.9788	0.42 ± 0.06	0.39 ± 0.06	1.0000
1/3DR‐BMD g/cm^2^	0.63 ± 0.07	0.64 ± 0.07	.8025	0.62 ± 0.05	0.61 ± 0.08	.0989
TR‐BMD g/cm^2^	0.50 ± 0.05	0.51 ± 0.07	.7510	0.52 ± 0.05	0.49 ± 0.06	.9778
LS‐T score	−1.34 ± 1.2	−1.20 ± 1.1	.0143	−1.14 ± 1.3	−1.56 ± 1.2	.0028
FN‐T score	−1.49 ± 1.1	−1.25 ± 1.0	.0003	−1.15 ± −1.3	−1.39 ± 1.0	.0077
TH‐ T score	−0.75 ± 0.9	−0.58 ± 1.0	.0027	−0.97 ± 1.0	0.72 ± 1.0	.1658
UDR‐T score	−0.80 ± 0.8	−0.63 ± 1.1	.7877	−0.47 ± 0.9	0.90 ± 1.0	.0942
1/3DR‐T score	−0.78 ± 1.0	−0.89 ± 1.2	.1289	−1.42 ± 1.2	−0.47 ± 0.9	.9523
TR‐T score	−0.94 ± 1.3	−1.25 ± 1.2	.8254	−0.97 ± 0.7	−1.68 ± 1.2	.9649
TBS	1.35 ± 0.13	1.29 ± 0.12	<.0001	1.35 ± 0.14	1.26 ± 0.13	<.0001

Note

Not performed.

Highlighted red values indicate statically significant values.

Abbreviations: 1/3 DR, 1/3 distal radius; BMD, bone mineral density; FN, femoral neck; LS, lumbar spine; TH, total hip; TR, total radius; UDR, ultradistal radius.

**FIGURE 1:**
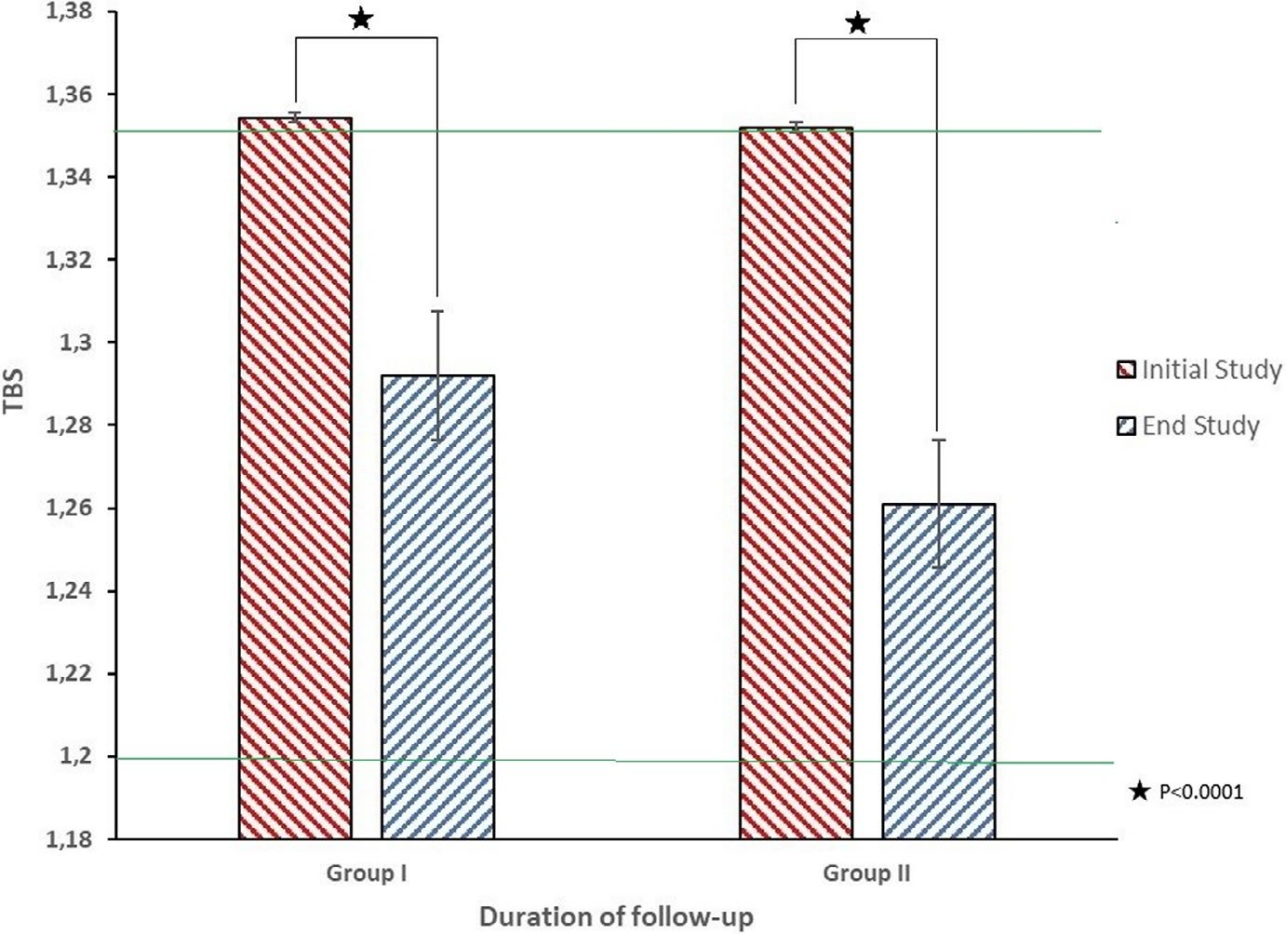
Baseline and end TBS scores according to the follow‐up of TSH suppression in patients of Group I (5‐10 years) and Group II (>10 years). Upper and lower lines indicates limits of normal, partially degraded and degrades TBS scores values

For patients in Group I, differences between absolute BMD at baseline and at the final study visit were not significantly different. However, a significant decrease in LS‐BMD (from 0.91 ± 0.18 g/cm^2^ to 0.87 ± 0.13 g/cm^2^, *P* = .0249) and FN‐BMD (from 0.78 ± 0.14 g/cm^2^ to 0.69 ± 0.11 g/cm^2^, *P* < .0001) was observed in patients of Group II. Reductions were also found in Group II T scores (LS‐T score from −1.14 ± 1.3 to −1.56 ± 1.2, *P* = .0028; FN‐T score from −1.15 ± 1.3 to −1.39 ± 1.0, *P* = .0077). TBS values were lower in both groups of compared with baseline values (Group 1, from 1.354 ± 0.131 to 1.289 ± 0.122, *P* < .0001; Group II from 1.354 ± 0.144 to 1.259 ± 0.129, *P* < .0001) (Figure 1). In Group I, there was a significant increase in T scores (LS‐T scores from −1.34 ± 1.2 to −1.20 ± 1.1, *P = *.0143; FN‐T scores from −1.49 ± 1.1 to −1.25 ± 1.0, *P* = .0003; and TH‐T scores from −0.75 ± −0.09 to −0.58 ± 1.0, *P *= .0027). The percent change from baseline was significantly different when comparing Group I to Group II for LS‐BMD (1.73 ± 9.3 vs 4.7 ± 79, *P* = .003), FN‐BMD (−0.15 ± 5.8 vs −12.2 ± 8.9, *P* < .0001), and TBS (−3.4 ± 6.6 vs −6.3 ± 9, *P* = .0213).

### 3.3 Stratification according to levels of TSH suppression

Clinical, thyroid hormone and bone parameters stratified by the level of TSH suppression at the final visit, are shown in Table 3. Although patients were treated in our Thyroid Cancer Unit by the same physician during follow‐up, there were changes in the degree of TSH suppression from the beginning to the end of the study, either due to intentional decision or from factors including age or BMI.

**TABLE 3:** Study of patients with DTC according to degree of TSH suppression

TSH suppression level (µIU/mL)	<0.1			0.1‐0.5			>0.5		
Parameters	Baseline (n = 75)	End study (n = 40)	*P*	Baseline (n = 48)	End study (n = 39)	*P*	Baseline (n = 22)	End study (n = 66)	*P*
Age (years)	51.7 ± 10.7	60.5 ± 9.8	<.0001	50.75 ± 12.7	62.62 ± 10.9	<.0001	52.26 ± 14.8	66.81 ± 10.4	.0001
BMI,(kg/m^2)^	27.38 ± 4.7	28.92 ± 5.8	.1705	26.93 ± 4.8	27.21 ± 4.8	.805	27.68 ± 5.1	28.92 ± 5.3	.3457
Serum TSH (µU/mL)	0.03 ± 0.01	0.03 ± 0.03	.9888	0.17 ± 0.1	0.25 ± 0.1	<.0001	1.16 ± 0.8	1.38 ± 0.9	.3511
Serum fT4, (ng/dL)	2.34 ± 0.5	1.85 ± 0.3	.1354	1.64 ± 0.5	1.68 ± 0.24	.3606	1.64 ± 0.4	1.54 ± 0.2	.5024
LT4 doses (mcg/Kg)	1.64 ± 0.4	1.75 ± 0.3	<.0001	2.24 ± 0.7	1.74 ± 0.34	<.0001	2.23 ± 0.6	1.59 ± 0.5	.0001
Duration (y) (range)	—	11.5 ± 5.9 (7‐21)		—	12.1 ± 6.3 (7‐21)			11.75 ± 5.9 (7.5‐18)	
Bone densitometry									
LS‐BMD, g/cm^2^	0.92 ± 0.14	0.91 ± 0.12	.5313	0.88 ± 0.19	0.88 ± 0.11	.6867	0.90 ± 0.15	0.88 ± 0.15	.5528
FN‐BMD, g/cm^2^	0.70 ± 0.13	0.72 ± 0.11	.5333	0.77 ± 0.15	0.69 ± 0.10	.0161	0.74 ± 0.13	0.69 ± 0.12	.3491
TH‐BMD, g/cm^2^	0.82 ± 0.09	0.87 ± 0.14	.0797	0.86 ± 0.13	0.86 ± 0.11	.9071	0.84 ± 0.10	0.84 ± 0.14	.7570
UDR‐BMD, g/cm^2^	0.50 ± 0.06	0.40 ± 0.07	.1514	0.41 ± 0.03	0.40 ± 0.05	.3609	0.38 ± 0.08	0.39 ± 0.06	.7410
1/3DR‐BMD, g/cm^2^	0.67 ± 0.08	0.63 ± 0.07	.5500	0.62 ± 0.05	0.63 ± 0.07	.6800	0.60 ± 0.07	0.61 ± 0.08	1.0000
TR‐BMD, g/cm^2^	0.59 ± 0.06	0.50 ± 0.09	.2320	0.52 ± 0.02	0.50 ± 0.06	.3765	0.49 ± 0.08	0.49 ± 0.07	.8688
LS‐T score	−1.13 ± 1.2	−1.25 ± 1.1	.5036	−1.35 ± 1.4	−1.51 ± 0.9	.6352	−1.35 ± 1.3	−1.44 ± 1.3	.7449
FN‐T score	−1.40 ± 1.0	−1.14 ± 1.02	.4563	−1.09 ± 1.4	−1.46 ± 0.9	.3039	−1.52 ± 1.2	−1.40 ± 1.1	.8725
TH‐ T score	−0.77 ± 0.8	−0.48 ± 0.9	.2839	−0.88 ± 1.1	−0.69 ± 0.9	.3541	−1.11 ± 0.8	−0.79 ± 1.1	.6018
UDR‐T score	0.96 ± 0.7	−0.73 ± 1.1	.1514	−0.49 ± 0.47	−0.81 ± 0.9	.3753	−1.17 ± 1.4	−0.95 ± 1.1	.7409
1/3DR‐T score	−0.36 ± 0.6	−1.10 ± 1.2	.5496	−1.32 ± 0.8	−1.02 ± 1.2	.6797	−1.53 ± 1.2	−1.4 ± 1.3	1.0000
TR‐T score	0.13 ± 0.3	−1.43 ± 1.2	.2312	−1.06 ± 0.4	−1.43 ± 1.1	.3452	−1.36 ± 1.1	−1.71 ± 1.2	.4736
TBS	1.36 ± 0.11	1.29 ± 0.13	.0086	1.34 ± 0.16	1.27 ± 0.12	.0855	1.32 ± 0.16	1.26 ± 0.13	.1463

Highlighted red values indicate statically significant values.

Abbreviations: 1/3 DR, 1/3 distal radius; BMD, bone mineral density; BMI, body mass index; FN, femoral neck; fT, free thyroxine; LS, lumbar spine; LT4, levothyroxine; Serum TSH, serum thyrotropin; TH, total hip; TR, total radius; UDR, ultradistal radius.

**FIGURE 2:**
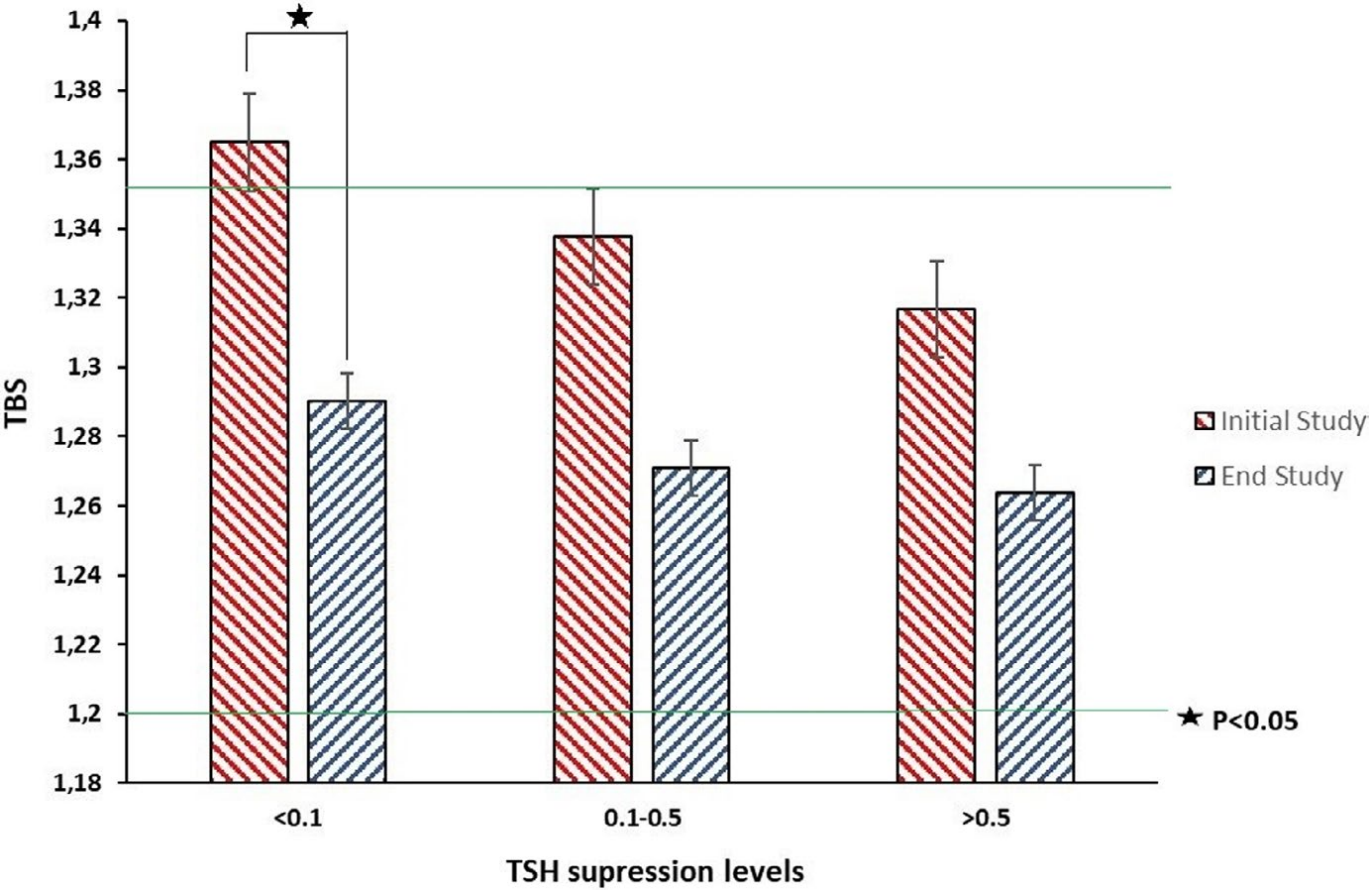
TBS scores of patients with DTC according to the degree of TSH suppression. Upper and lower lines indicates limits of normal, partially degraded and degrades TBS scores values

During the course of follow‐up, there was a reduction in the number of patients with suppressed TSH (<0.1 µU/mL) and moderately suppressed TSH (0.1‐0.5 µU/mL), while the number of patients with nonsuppressed TSH (>0.5 µU/mL) increased following the guidelines of DTC. The duration of TSH suppression in the three groups was similar. No significant changes in BMI were observed in any of the TSH suppression levels analyzed. The percent change from baseline in BMD was −3.2 ± 9.2 for LS‐BMD, −6.7 ± 7.6 for FN‐BMD, −4.2 ± 17 for TH‐BMD, and −3.4 ± 7.9 for TBS in patients with suppressed TSH. In patients with moderately suppressed TSH, percent change from baseline was −3.2 ± 12 for LS‐BMD, −10.2 ± 11.4 for FN‐BMD, −1.2 ± 9.2 for TH‐BMD, and −5.2 ± 10.4 for TBS. For patients with nonsuppressed TSH, percent change from baseline was 11.3 ± 84 for LS‐BMD, −4.3 ± 8.7 for FN‐BMD, 2.2 ± 7.6 for TH‐BMD, and −4.7 ± 7 for TBS. TBS was significantly reduced in patients with TSH suppression <0.1 µU/mL (from 1.362 ± 0.112 to 1.289 ± 0.132, *P* = .0086), whereas only nonstatistically significant reductions in TBS were seen in patients with lower levels of TSH suppression (Figure 2).

In the final visit the number of patients from the Group with TSH suppression <0.1 µU/mL, that continued with this suppressed values, was reduced to 26. This subgroup had low TBS values compared to initial suppressed group values (n = 75) (1.278 ± 0.130 vs, 1.362 ± 0.112, *P* = .0056). Taking in consideration the subgroup of 49 patients that change from total suppression, to moderate or no suppression, they also low values of TBS (1.272 ± 0.103) at the final visit study, as well as those 70 that continued to maintain the initial moderate or no suppression, with low TBS values (1.26 ± 0.14, *P* = .0062). According to the grade of risk of TBS values in the initial study there were: 23 patients (15.9%) with TBS < 1.23 (degraded); 45 (31%) with a TBS score between 1.23 and 1.35 (partial degraded), and 77 (53.1%) with TBS > 1.35 (normal); and the final study there were 35 patients (24.1%) with TBS < 1.23(degraded), 72 (49.65%) with a TBS score between 1.23 and 1.35 (partial degraded), and 38 (26.2%) with TBS > 1.35 (normal). At the final study, there was an important increase in the number of patients with degraded and partially degraded microarchitecture, while there was a decrease in more than a half, in patients with normal TBS scores and normal LS‐BMD T scores (42% vs 13.3%)(Fisher´s exact test < 0.0001).

Pearson correlation demonstrated a significant positive correlation between TBS with BMD at all analyzed sites: LS‐BMD (*r* = .35, *P* = .0001); FN‐BMD, (*r* = .33, *P* = .0004); TH, (*r* = .25, *P* = .0076); 1/3 DR, (0.49, *P *< .0001), but we did not find correlations between TBS and other studied parameters including duration (*P* = .8006), TSH suppression levels (*P* = .1293), or serum fT4 levels (*P* = .1100) at the final study visit. Neither duration of suppression (*P* = .6220), serum fT4 (*P* = .9207) nor TSH levels (*P* = .3920) were correlated with LS‐BMD. The three levels of TSH suppression were also not correlated with BMD.

In the multivariate analysis, clinical parameters associated with TBS as a dependent variable were: levels of TSH suppression below 0.1 µU/mL (*B* = 0.2542, *P* < .0280); between 0.1 and 0.5 µU/mL (*B* = 0.2271, *P* = .0513), and >0.5 µU/mL (*B* = 0.2327, *P* = .0427). Adjustments for BMD were made to show that the association between TBS and duration of TSH suppression was independent from BMD.

## 4 DISCUSSION

Our study provide information that total thyroidectomized female patients due to DTC who received long‐term TSH suppressive therapy had lower vertebral TBS, after both 5‐10 years and >10 years of follow‐up. In contrast, LS‐BMD and FN‐BMD decreased significantly only in patients receiving TSH suppression for >10 years. This suggests that under TSH suppression therapy trabecular bone structure could be damaged even before changes in BMD are apparent.

Our data showed that DCT patients with <10 years of follow‐up had BMD in the normal range, while there were abnormalities in TBS, suggesting that this parameter could provide a more sensitive assessment of bone health in these patients. Recently, in a retrospective cross‐sectional study, Moon et al showed that 4.2 years of TSH suppression therapy in postmenopausal patients with DTC was associated with a significant decrease in TBS, independent of BMD changes.^21^ In a previous study by the same authors, TSH suppression of 3.8 ± 1.2 years was associated with decreased bone strength by altering hip bone geometry rather than BMD.^22^ Our group has also shown, in 84 postmenopausal women with DTC, a significant reduction in TBS although the different degree neither years of TSH suppression therapy was not analyzed.^23^ In the present study, TBS values were similar when analyzed in postmenopausal women alone without the premenopausal group.

The present study suggests that TBS may be an earlier marker of bone abnormalities than BMD analysis, and may provide for earlier indication of fracture risk in these patients. Moderate suppression of TSH in the range of 0.1‐0.5 µIU/mL, and of nonsuppression >0.5 µIU/mL, were associated with lower values of TBS, and should be observed during the life‐long treatment of these patients. In our study serum fT4 levels were not associated with TBS, reinforcing the role of TSH in the deterioration of bone microstructure. Also, recently in premenopausal women with nontreated Graves´ disease, high serum fractaline (chemokine CX3CL1), and lower TBS were found, indicating microarchitectural deterioration linked to increased bone remodeling in these patients.^24^ This study support the fact that TBS could be early marker of bone deterioration, and thus, of fracture risk in these low TSH levels patients.

The relationship between excess exogenous thyroid hormone, serum TSH and bone mass are conflicting.^25^ In a recent study of 93 989 patients newly diagnosed with thyroid cancer, the cumulative duration of LT4 use was associated with a 3.3‐fold higher risk of osteoporosis after 7.5 years of duration. However, that study did not report bone densitometry for definition of bone loss, smoking prevalence, body weight, or TSH values.^26^ Greater bone loss has been described in patients with DTC, who have suppressed TSH levels when compared to nonsuppressed patients,^27^ and it has been suggested, that the greater risk of major osteoporotic fractures in hypothyroid patients could be driven by periods of low TSH from excessive thyroid replacement.^28^ Moreover, suppressive doses of LT4 could also induce iatrogenic subclinical hyperthyroidism, and therefore, subsequently enhance bone resorption. Reduction of TSH levels below 0.1 µU/mL increased by 3‐4.5 the risk of vertebral and nonvertebral fractures.^29^ Although we did not capture the incidence of fractures in this study cohort, significant reductions from baseline in TBS were only seen in the subpopulation with suppressed TSH (levels < 0.1 µU/mL), which may suggest the use of TBS as a proxy for fracture risk.

We did not find differences in dietary calcium intake, smoking rates or physical activity between the studied groups. The average calcium intake was 575.9 ± 282 mg/day and there were no differences in the intake among the three studied TSH suppression groups. This value is similar to the mean calcium dietary intake (698 ± 313 mg/day) reported in a large Spanish population study.^30^ There was a modest negative correlation between BMI and TBS (*r* = −.17), whereas BMI was positively correlated with LS.BMD (*r* = .30). Increase in adiposity overlying the Region of Interest (ROI) may lower the signal‐to‐noise ratio, favoring a lower TBS. In our study, BMI patients BMI was well within the working range recommended for TBS (15‐37 kg/m^2^)^31^. The use of older TBS software version 1.8 gave lower values for men than for women, while the new update versions are less affected by BMI.^32^

Our study has a number of clinical implications. In patients with thyroid cancer and long‐term TSH suppression treatment, microarchitecture deterioration can be found in many of them, and risk of osteoporosis and fractures should be evaluated with TBS and DXA. Our results highlight the importance of trabecular analysis. Major strength of our study include, that patients were followed in a single center. This study has the following limitations: the absence of a control group without LT4, a treatment that cannot be denied for ethical reasons; the lack of information regarding the incidence of fractures during TSH suppression; and the potential impact of menopause status. Also the studied was made in a homogenous population (Caucasian women), and thus, we cannot generalize results to other populations. The key strength of this study was that patients were followed up for a long duration in the Thyroid Unit of a single center, and all DXA analyses were performed with the same equipment.

In conclusion assessment of TBS in patients with DTC, who underwent a total thyroidectomy and have long‐term TSH suppression therapy, can reveal a deterioration of trabecular bone, and confirms the utility of this technique for evaluating skeletal fragility and potential fracture risk. Unlike TBS, we did not find significant changes in BMD when patients were stratified by TSH level. Our study indicates that TSH suppression levels can be a major factor in TBS and BMD deterioration over long‐term follow‐up on these patients. Further studies are necessary to determine the adequate levels of thyroid hormone and TSH levels that do not deteriorate bone quality in these patients.

## CONFLICT OF INTEREST

The authors declare no conflicts of interest have nothing to disclose.

## AUTHOR CONTRIBUTIONS

Study design: FH and MD. Study conduct: MM, BL. Data acquisition: MD. Data analysis: CA. Data interpretation: FH, CA, SG. Drafting manuscript: FH, GA, GM. Revising manuscript content and approving final version of the manuscript: FH, GA.

## Data Availability

The data that support the findings of this study are available from the corresponding author upon reasonable request.

## References

[1] Chen AY , Jemal A , Ward EM . Increasing incidence of differentiated thyroid cancer in the United States 1988–2005. Cancer. 2009;115:3801‐3807. 19598221 10.1002/cncr.24416

[2] Freudenthal B , Williams GR . Thyroid stimulating hormone suppression in the long‐term follow‐up of differentiated thyroid cáncer. Clin Oncol. 2017;29:325‐328. 10.1016/j.clon.2016.12.011 28043744

[3] Haugen BR , Alexander EK , Bible KC , et al. 2016 American thyroid association management guidelines for adult patients with thyroid nodules and differentiated thyroid cancer. The American Thyroid Association Guidelines Task Force on Thyroid Nodules and Differentiated Thyroid Cancer. Thyroid. 2016;26:1–133. 26462967 10.1089/thy.2015.0020 PMC4739132

[4] McGriff NJ , Csako G , Gourgiotis L , et al. Effects of thyroid hormone suppression therapy on adverse clinical outcomes in thyroid cancer. Ann Med. 2002;34:554‐564. 12553495 10.1080/078538902321117760

[5] Jonklaas J , Sarlis NJ , Litofsky D , et al. Outcomes of patients with differentiated thyroid carcinoma following initial therapy. Thyroid. 2006;16:1229‐1234. 17199433 10.1089/thy.2006.16.1229

[6] Sugitani I , Fujimoto Y . Does postoperative thyrotropin suppression therapy truly decrease recurrence in papillary thyroid carcinoma? A randomized controlled trial. J Clin Endocrinol Metab. 2010;95:4576‐4583. 20660039 10.1210/jc.2010-0161

[7] Leese GP , Jung RT , Guthrie C , Waugh N , Browning MCK . Morbidity in patients on L‐thyroxine: a comparison of those with a normal TSH to those with a suppressed TSH. Clin Endocrinol. 1992;37:500‐503. 10.1111/j.1365-2265.1992.tb01480.x 1286519

[8] Lee JS , Buzková P , Fink HA . Subclinical thyroid dysfunction and incident hip fracture in older adults. Arch Intern Med. 2010;170:1876‐1883. 21098345 10.1001/archinternmed.2010.424 PMC4122328

[9] Garin MC , Arnold AM , Lee JS , Robbins CAR . Subclinical thyroid dysfunction and hip fracture and bone mineral density in older adults: the cardiovascular health study. J Clin Endocrinol Metab. 2014;99:2657‐2664. 24878045 10.1210/jc.2014-1051 PMC4121038

[10] Flynn RW , Bonellie SR , Jung RT , et al. Serum thyroid‐stimulating hormone concentration and morbidity from cardiovascular disease en fractures in patients on long‐term thyroxine therapy. J Clin Endocrinol Meab. 2010;95:186‐193. 10.1210/jc.2009-1625 19906785

[11] Blum MR , Bauer DC , Collet TH , et al. Subclinical thyroid dysfunction and fracture risk. A meta‐analysis. JAMA. 2015;313:2055‐2065. 26010634 10.1001/jama.2015.5161 PMC4729304

[12] ZhuH ZhangJ , WangJ ZX , Gu M . Association of subclinical thyroiddysfunction with bone mineral density and fractures: a meta‐analysis of prospective cohort studies. Endocrine. 2020;67:685‐698. 31721088 10.1007/s12020-019-02110-9

[13] Biondi B , Cooper DS . Benefits of thyrotropin suppression versus the risks of adverse effects in differentiated thyroid cancer. Thyroid. 2010;20:135‐146. 20151821 10.1089/thy.2009.0311

[14] Papaleontiou M , Banerjee M , Reyes‐Gastelum D , Hawley ST , Haymart MR . Risk of osteoporosis and fractures in thyroid cancer: a case‐control study in US veterans. Oncologist. 2019;24:1166‐1173. 31164453 10.1634/theoncologist.2019-0234 PMC6738319

[15] Papaleontiou M , Hawley ST , Haymart MR . Effect of thyrotropin suppression therapy on bone in thyroid cancer patients. Oncologist. 2016;21:165‐171. 26659220 10.1634/theoncologist.2015-0179 PMC4746080

[16] Bousson V , Bergot C , Sutter B , et al. Trabecular bone score (TBS): available knowledge, clinical relevance, and future prospects. Osteoporos Int. 2012;23:1489‐1501. 22083541 10.1007/s00198-011-1824-6

[17] Hans D , Stenover E , Larry O . The trabecular bone score (TBS) complements DXA and the FRAX as for risk assessment tool in routine clinical practice. Curr Osteoporous Rep. 2017;15:521‐523. 10.1007/s11914-017-0410-z 28988401

[18] Kanis JA , Melton LJ , Christiansen C , et al. The diagnosis of osteoporosis. J Bone Miner Res. 1994;9:1137‐1141. 7976495 10.1002/jbmr.5650090802

[19] Diaz Curiel M , Carrasco de la Peña JL , Honorato Perez J , et al. Study of bone mineral density in lumbar spine and femoral neck in a Spanish population. Multicentre Research Project on Osteoporosis. Osteoporos Int. 1997;7:59‐64. 9102065 10.1007/BF01623462

[20] Cormier C , Lamy O , Poriau S . TBS in routine clinical practice: proposal of use. [internet]. Plan.les‐Outes, Switzerland: Medimaps Group, 2012 Available from: http://www.medimapsgroup.com/upload/MEDIMAPS‐UK‐WEB.pdf

[21] Moon JH , Kim KM , Oh TJ , et al. The effect of TSH suppression on vertebral trabecular bone scores in patients with differentiated thyroid carcinoma. J Clin Endocrinol Metab. 2017;102:78‐85. 27754806 10.1210/jc.2016-2740

[22] Moon JH , Jung KY , Kim KM , et al. The effect thyroid stimulating hormone suppressive therapy on bone geometry in the hip area of patients with differentiated thyroid carcinoma. Bone. 2015;83:104‐110. 26518742 10.1016/j.bone.2015.10.015

[23] De Mingo ML , Guadalix S , Arriscado M , et al. Low trabecular bone score in postmenopausal women with differentiated thyroid carcinoma after long‐term TSH suppressive therapy. Endocrine. 2018;62:166‐173. 30014437 10.1007/s12020-018-1671-8

[24] Kuzma M , Vnuga P , Binkley N , et al. High serum Fractalkine is associated with lower trabecular bone score in premenopausal women with Grave´s disease. Horm Metab Res. 2018;50:609‐614. 29954010 10.1055/a-0633-2814

[25] Biondi B , Cooper DS . Thyroid hormone suppression therapy. Endocrinol Metab Clin North Am. 2019;48:227‐237. 30717904 10.1016/j.ecl.2018.10.008

[26] Lin SY , Lin CL , Chen HT , et al. Risk of osteoporosis in thyroid cancer patients using levothyroxine: a population‐based study. Curr Med Res Opin. 2018;34:805–812. 28884595 10.1080/03007995.2017.1378174

[27] Kim MK , Yun KJ , Kim MH , et al. The effects of thyrotropin suppressing therapy on bone metabolism in patients with well differentiated thyroid carcinoma. Bone. 2015;2015:101‐105. 10.1016/j.bone.2014.10.009 25445448

[28] Abrahamsen B , Jørgensen HL , Laulund AS , et al. The excess risk of major osteoporotic fractures in hypothyroidism is driven by cumulative hyperthyroid as opposed to hypothyroid time: an observational register‐based time‐resolved cohort analysis. J Bone Miner Res. 2015;30:898‐905. 25431028 10.1002/jbmr.2416

[29] Bauer DC , Ettinger B , Nevitt MC , Stone KL ; Study of Osteoporotic Fractures Research Group . Risk for fracture in women with low serum levels of thyroid‐stimulating hormone. Ann Intern Med. 2001;134:561‐568. 12803168 10.7326/0003-4819-134-7-200104030-00009

[30] Olza J , Aranceta‐Bartrina J , Reported G‐G , et al. Dietary intake, disparity between the reported consumption and the level needed for adequacy and food sources of calcium, phosphorus, magnesium and vitamin D in the Spanish population: findings from the ANIBES Study. Nutrients. 2017;9:168. 10.3390/nu9020168 PMC5331599 28230782

[31] McCloskeyEV OA , Harvey NC , Leslie WD , et al. A meta‐analysis of trabecular bone score in fracture prediction and its relationship to FRAX. JBMR. 2016;31:940‐948. 10.1002/jbmr.2734 26498132

[32] Schacter GI , Leslie WD , Majumdar SR , Morin SN , Lix LM , Hans D . Clinical performance of an updated trabecular bone score (TBS) algorithm in men and women: the Manitoba BMD cohort. Osteoporos Int. 2017;283:3199‐3203. 10.1007/s00198-017-4166-1 28733715

